# Prevalence and characteristics of COVID-19 vaccine breakthrough infection in Upper Egypt

**DOI:** 10.1186/s43168-023-00196-4

**Published:** 2023-04-21

**Authors:** Aliae A. R. Mohamed Hussein, Maiada K. Hashem, Mohammed G. Azizeldine, Ahmad M. Shaddad

**Affiliations:** Chest Department, Assiut University Hospital, Assiut Faculty of Medicine, Assiut University, Assiut, 71515 Egypt

**Keywords:** COVID-19, Breakthrough, COVID-19 vaccination, SARS-COV-2

## Abstract

**Background:**

Infection breakthrough after COVID-19 vaccination is a point of conflict in current literature not only because of the estimation of the potential hazards and symptoms severity but also due to establishing a vaccination policy and measuring the extent of immunity after vaccination in addition to the waning of the humoral immunity over time. To our knowledge, this is the first study to stratify the risk of post-COVID-19 vaccination breakthroughs in Upper Egypt.

**Methods:**

In this cross-sectional observational study, we enrolled 369 vaccinated patients registered in our facility either admitted or in out-patient clinic. Patients were interviewed and any proven history of COVID-19 infection after vaccination was recorded.

**Results:**

In the current study, 18.97% (70 patients) of the 369 subjects enrolled in the study had COVID-19breakthrough infection. Students were the high-risk group representing 18.6% of the study subjects. Hypertension, diabetes, and cardiac disease were the most comorbidities associated with a prevalence of 15.7%, 8.6%, and 4.3% respectively. Prior to COVID-19, confirmed infection was present in 42.9% of the study group. Fever, headache, myalgia, and cough were among the most common symptoms of the post-COVID vaccination breakthrough with a prevalence of 90%, 75.7%, 84.3%, and 74.3% respectively.

**Conclusion:**

Breakthrough infection after COVID-19 vaccination is not uncommon. Most cases are mild and don’t require hospitalization. All types of vaccines tested in the current study offer adequate immunity and guard against severe COVID-19 infection. We encourage the current global policy of full vaccination.

**Trial registration:**

ClinicalTrials.gov. NCT05033834. Registered September 5, 2021. COVID-19 Infection in After Vaccination-Full Text View-ClinicalTrials.gov.

## Introduction

Coronavirus disease 2019 (COVID-19), caused by severe acute respiratory syndrome coronavirus 2 (SARS-CoV-2), was first identified in Wuhan, China [1]. It rapidly spread, resulting in a global pandemic in March 2020. Globally, until 27 August 2021, there have been over 214 million confirmed cases of COVID-19, including more than 4 million deaths reported to WHO [2].

With the rapid and disastrous spread of COVID-19 infection and the absence of specific antiviral treatment, it was mandatory for the world to collaborate and develop vaccines. The Pfizer/BioNTech Comirnaty vaccine was listed for WHO Emergency Use Listing (EUL) on 31 December 2020. The SII/Covishield and AstraZeneca/AZD1222 vaccines (developed by AstraZeneca/Oxford and manufactured by the State Institute of India and SK Bio respectively) were given EUL on 16 February. The Janssen/Ad26.COV 2.S developed by Johnson & Johnson, was listed for EUL on 12 March 2021. The Moderna COVID-19 vaccine (mRNA 1273) was listed for EUL on 30 April 2021 and the Sinopharm COVID-19 vaccine was listed for EUL on 7 May 2021. The Sinopharm vaccine is produced by Beijing Bio-Institute of Biological Products Co Ltd., China National Biotec Group (CNBG) subsidiary. The Sinovac-CoronaVac was listed for EUL on 1 June 2021. From 25 August 2021 till the end of 2022, over 5 billion worldwide received two doses of one of the COVID-19 vaccines. In large, randomized-controlled trials, vaccines were found to be safe and efficacious in preventing symptomatic, laboratory-confirmed COVID-19 [3–5].

However, a small percentage of fully vaccinated persons will develop symptomatic or asymptomatic infections with SARS-CoV-2, which causes COVID-19. A vaccine breakthrough infection is defined as the identification of SARS-CoV-2 RNA or antigen in a respiratory tract sample collected from a person ≥ 14 days after receipt of all recommended doses of an FDA-authorized COVID-19 vaccine. By the end of April 2021, there were more than 10,000 vaccines for breakthrough infection. 63% of those cases were females and the median age of reported cases was 58. Regarding stratification of the risk and severity of the vaccine breakthrough infection, studies showed that 27% of those infections were asymptomatic and 10% of cases needed hospitalization and death occurred in 2% of cases [6].

As it is a recently manifest problem, knowledge and statistics about vaccine breakthroughs are still limited in Egypt. According to this perspective, the current study aims to spotlight the vaccine breakthrough infection prevalence, pattern, and severity in Upper Egypt.

## Patients and methods

### Study design

This cross-sectional observational study including COVID-19 vaccinated cases has been conducted at ICU, inpatient ward, outpatient clinic, post-COVID-19 clinics, and Vaccination Unit, Chest Department, Assiut University hospitals from the 1st of September 2021 to the end of March 2022.

### Study subjects, inclusion, and exclusion criteria

We enrolled 369 subjects in this study either from patients admitted to the hospital or the outpatient clinic. Patients diagnosed with Breakthrough COVID-19 either based on symptoms and HRCT and/or PCR. All subjects of 18 years old and above of both genders received at least one dose of COVID-19 vaccines available and were diagnosed COVID-19 positive after vaccination by real-time PCR (confirmed case) or combined clinical and radiological diagnosis (possible case) were illegible to participate in this study. Exclusion criteria were children less than 18 years and patients who did not receive COVID-19 vaccination.

### Data collection

The recruited cases were divided into two groups:

#### Group A

People receiving vaccination and did not develop new COVID-19 infection (breakthrough) during the study period.

#### Group B

Patients who developed recent COVID-19 infection after at least one dose of vaccination.

All subjects were interviewed and these data were collected: personal history, medical history, any comorbid condition, medications, history of other vaccines, history of COVID-19 vaccination, type of vaccine, date of vaccination, and members of doses received, history of recent COVID-19 infection, date of infection, time lag between vaccination and severity of infection; symptomatology, radiology, need for hospitalization, need for oxygen therapy, or need for ICU admission.

Patients will be classified according to the severity of infection: mild, moderate, severe, and critical according to Ministry of Health Guidelines (MoH) updates-version 6, 2021 [7] and also will be classified according to the time lag between vaccination and infection to < 14 days after the first dose, > 14 days after the first and before the second dose, < 14 days of the second dose (post-vaccine infection in all types except Johnson single dose vaccine), and after 14 days of full vaccination (breakthrough).

### Real-time PCR

A group of patients was diagnosed with COVID-19 according to PCR 7–500 FAST made in the USA.

### Chest HRCT in COVID-19

The computed tomography was performed using GE Optima 64 slices made in the USA. Patients were examined in the supine position and the scanning range included the whole chest from the thoracic inlet down to the diaphragm. MSCT of the chest was observed for signs of recent COVID-19 infection and diagnosis and severity were assessed using 5 grades CO-RADS system.

### Statistical analysis

Data was collected and analyzed by using SPSS (Statistical Package for the Social Science, version 20, IBM, and Armonk, New York). The Shapiro test was used to determine the compliance of the data to normal distribution. Quantitative data with abnormal distribution were expressed as median (range) and compared to the Kruskall-Wallis test while normally distributed data was expressed as mean (SD) and compared by Student’s *t* test.

Nominal data were given as a number (*n*) and percentage (%). The chi2 test was implemented on such data. Logistic regression analysis was used to determine independent predictors for developing post-vaccine COVID-19 infection. Pearson coefficient correlation determined correlations between different continuous variables in the study. The correlation between continuous and nominal variables was assessed by Eta value while the correlation between two nominal variables was assessed by Crammer’s *V* test. The level of confidence was kept at 95% and hence, the *P* value was considered significant if < 0.05.

## Results

The mean age of patients with post-vaccine COVID-19 infection was significantly higher in comparison to those who did not develop infection (37.17 ± 17.97 vs. 27.66 ± 12.29 (years); *p* < 0.001). More than half of the participants of both groups were males and came from urban areas with no significant differences. The majority (67.1%) of those who developed post-vaccine COVID-19 infection were employees while (54.8%) of those without infection were students with a significant difference between both groups (*p* < 0.001) (Table 1).

**Table 1:** Sociodemographic data of COVID-19 vaccinatedAQ3 cases included in the study (*n* = 369)

	Post-vaccine COVID-19		***P*** value*
	Group A (***n***= 299)	Group B (***n***= 70)	
Age (years)	27.66 ± 12.29	37.17 ± 17.97	**< 0.001**
Sex			
Male	164 (54.8%)	39 (55.7%)	0.50
Female	135 (45.2%)	31 (44.3%)	
Residence			
Rural	141 (47.2%)	33 (47.1%)	0.55
Urban	158 (52.8%)	37 (52.9%)	
Marital status			
Married	84 (28.1%)	32 (47.1%)	**0.01**
Single	215 (71.9%)	37 (52.9%)	
Occupation			
Employee	99 (33.1%)	47 (67.1%)	**< 0.001**
Worker	8 (2.7%)	5 (7.1%)	
None	28 (9.4%)	5 (7.1%)	
Student	164 (54.8%)	13 (18.6%)	

Data expressed as frequency (percentage), mean (SD). *P* value was significant if < 0.05. **COVID-19**: coronavirus disease-19

*Age was compared with Student t test while all other data were compared by *Chi*^*2*^ test

Patients with post-vaccine COVID-19 infection had a significantly higher frequency of hypertension (15.7% vs. 2.3%; *p* < 0.001), diabetes mellitus (8.6% vs. 2.3%; *p* = 0.02) and cardiac disease (4.3% vs.0; *p* < 0.001) in comparison to those without post-vaccine COVID-19 infection. Both groups had insignificant differences regarding renal diseases, chest diseases, and other comorbidities (Table 2).

**Table 2:** Comorbidities among group A patients who did not developed post-vaccine COVID-19 infection (*n* = 299) versus group B vaccinated participants who developed post-vaccine COVID-19 infection (*n* = 70)

	COVID-19 vaccinated cases (*n* = 369)		*P* value*
	Group A (*n* = 299)	Group B (*n* = 70)	
Hypertension	7 (2.3%)	11 (15.7%)	** < 0.001**
Diabetes mellitus	7 (2.3%)	6 (8.6%)	**0.02**
Cardiac diseases	0	3 (4.3%)	** < 0.001**
Renal diseases	1 (0.3%)	1 (1.4%)	0.34
Chest diseases	2 (0.7%)	2 (2.9%)	0.16
Others^a^	2 (0.7%)	2 (2.9%)	0.16

Data expressed as frequency (percentage). Only significant values should be in boldface (*P* value was considered significant if < 0.05). *COVID-19* coronavirus disease-19

^*^All data were compared by *Chi*^*2*^ test

^a^Others include hyperthyroidism, cardiovascular diseases, gout, peptic ulcer, osteoarthritis, and typhoid fever

The frequency of prior COVID-19 infection was significantly higher among patients who developed post-vaccine COVID-19 infection (*P* = 0.001). The severity of the prior infection was mild, moderate, and severe in 10 (19.2%), 23 (44.2%) and 19 (36.5%) among patients with post-vaccine infection, and 4 (13.3%), 19 (63.3%), and 7 (23.3%) among those with post-vaccine infection, respectively (Table 3).

**Table 3:** Characteristics of COVID-19 infection prior to the vaccine in recruited cases (*n* = 369)

	COVID-19 vaccinated cases (*n* = 369)		*P* value*
	Group A (*n* = 299)	Group B (*n* = 70)	
Duration before vaccination (days)	158 (8–670)	160 (19–466)	0.53
Diagnostic symptoms^a^	52 (94.5%)	29 (96.7%)	0.55
Diagnostic CT chest^b^	13 (23.6%)	4 (13.3%)	0.19
Severity			0.24
Mild	10 (19.2%)	4 (13.3%)	
Moderate	23 (44.2%)	19 (63.3%)	
Severe	19 (36.5%)	7 (23.3%)	
Prior infection			** < 0.001**
Yes	55 (18.4%)	30 (42.9%)	
No	244 (81.6%)	40 (57.1%)	

Data expressed as frequency (percentage), median (range). Only significant values should be in boldface (*P* value was considered significant if < 0.05). *COVID-19* coronavirus disease-19, *CT* computed tomography

^*^Duration before vaccine was compared by Mann Whitney test while all other data were compared with *Chi*^*2*^ test

^a^Include fever, myalgia, ageusia, anosmia, cough, diarrhea, rhinorrhea, sore throat, and dyspnea

^b^CORAD 3 to CORAD 5

The duration between vaccination and occurrence of COVID-19 infection ranged between 14 and 303 days with a median duration of about 41 days. Thirty-seven (52.9%) patients developed infection > 28 days after the 2nd dose (vaccine breakthrough) while 19 (27.1%), 8 (11.4%), and 6 (8.6%) patients developed infection > 14 days after the first dose, > 28 days of first dose and > 14 days the of the second dose, respectively.

Twenty-two (31.4%) patients were diagnosed using swabs for PCR while 21 (30%) patients were diagnosed by typical radiological criteria in CT chest.

With the exception of only one patient, all patients with post-vaccine COVID-19 infection had various symptoms. The most frequently reported symptoms were fever (90%), myalgia (84.3%), headache (75.7%), and cough (74.3%). Other symptoms are summarized in (Table 4).

**Table 4:** Pattern of post-vaccine COVID-19 infection in group B (*N* = 70)

	*N* = 70
Frequency according to duration of infection	
> 14 days of 1st dose	19 (27.1%)
> 28 days of 1st dose	8 (11.4%)
> 14 days of 2nd dose	6 (8.6%)
> 28 days of 2nd dose	37 (52.9%)
Duration from vaccine to infection (days)	41 (14–303)
Diagnostic swab for PCR	22 (31.4%)
Diagnostic chest HRCT^a^	21 (30%)
Diagnostic symptoms^b^	69 (98.6%)
Fever	63 (90%)
Headache	53 (75.7%)
Myalgia	59 (84.3%)
Ageusia	18 (25.7%)
Anosmia	20 (28.6%)
Cough	52 (74.3%)
Diarrhea	7 (10%)
Rhinorrhea	21 (30%)
Sore throat	28 (40%)
Dyspnea	32 (45.7%)

Data expressed as frequency (percentage), median (range). *COVID-19* coronavirus disease-19, *HRCT* high-resolution computed tomography

^a^CORAD 3 to CORAD 5

^b^Include fever, myalgia, ageusia, anosmia, cough, diarrhea, rhinorrhea, sore throat, and dyspnea

Up to 71% of patients with post-vaccine COVID-19 infection required only home isolation while only one patient required home oxygen therapy. Twelve patients (17.1%) were admitted to the hospital ward while the other 7 (10%) required intensive care unit admission (Table 5).

**Table 5:** Site of care for patients developed post-vaccine COVID-19 infection group B (*N* = 70)

Care site of treatment	*N* = 70
Home isolation	50 (71.4%)
Home oxygen therapy	1 (1.4%)
Hospital admission	12 (17.1%)
Intensive care unit isolation	7 (10%)

Data expressed as frequency (percentage). *COVID-19* coronavirus disease-19

There were significant differences between patients who developed post-vaccine COVID-19 infections and participants who did not develop infection regarding the type of vaccine. The most frequently used vaccines in the case of those with post-vaccine infection were AstraZeneca (44.3%), Sinopharm (22.9%), and Sinovac (20%) while the most frequently used vaccines in those without post-vaccine infection were Sinovac (36.8%), Sinopharm (29.1%) then AstraZeneca (25.4%). None of those who developed post-vaccine COVID-19 infection received the Moderna vaccine.

Although the majority of subjects in both groups were fully vaccinated, the frequency of fully vaccinated subjects was significantly lower in those who developed a post-vaccine infection (75.7% vs. 89%; *p* < 0.001) compared to those without post-vaccine COVID-19 infection (Table 6). Different types of vaccines showed no significant differences regarding different patterns of post-vaccine COVID-19 infection with the exception of duration between vaccination and diagnosis of infection which was significantly shorter among those who received the Sinovac vaccine (22 days) and Pfizer vaccine (31 days) but it was longer among those who received Johnson (85 days).

**Table 6:** Type of vaccine and doses used among patients developed post-vaccine COVID-19 infection (group B) versus vaccinated participants who did not develop infection group A

	COVID-19 vaccinated cases (*n* = 369)		*P* value*
	Group A (*n* = 299)	Group B (*n* = 70)	
Type of vaccine			** < 0.001**
AstraZeneca	76 (25.4%)	31 (44.3%)	
Sinofarm	87 (29.1%)	16 (22.9%)	
Sinovac	110 (36.8%)	14 (20%)	
Johnson	5 (1.7%)	1 (1.4%)	
Pfizer	15 (5%)	5 (7.1%)	
Moderna	5 (1.7%)	0	
Sputnik	1 (0.30%)	3 (4.3%)	
Dosing			** < 0.001**
Partially vaccinated	33 (11%)	17 (24.3%)	
Fully vaccinated	266 (89%)	53 (75.7%)	

Data expressed as frequency (percentage). Only significant values should be in boldface (*P* value was considered significant if < 0.05). *COVID-19* coronavirus disease-19

^*^All data were compared with *Chi*^*2*^ test

Regarding the present symptoms, diarrhea was the only symptom with statistically significant differences between the different groups (*p* = 0.04). It was absent in those who received Sinovac and Johnson vaccines and more frequently among those who received Pfizer (40%) Sputnik (33.3%) and less frequently among those who received AstraZeneca (3.2%) Sinopharm (18.8%) vaccines. Despite other symptoms showing no significant statistical difference, dyspnea showed a noticeable difference between different groups (*p* = 0.06) (Table 7).

**Table 7:** Pattern of post-COVID-19 infection based on types of vaccine (*N* = 70)

	AstraZenca	Sinofarm	Sinovac	Johnson	Pfizer	Sputnik	*P*
Vaccination							
Partial	5 (16.1%)	3 (18.8%)	6 (42.9%)	0	2 (40%)	1 (33.3%)	
Fully	26 (83.9%)	13 (81.3%)	8 (57.1%)	1 (100%)	3 (60%)	2 (66.7%)	0.39
Incidence							
> 14 days of 1st dose	4 (12.9%)	4 (25%)	9 (64.3%)	0	1 (20%)	1 (33.3%)	
> 28 days of 1st dose	5 (16.1%)	1 (6.3%)	0	0	2 (40%)	0	0.09
> 14 days of 2nd dose	5 (16.1%)	1 (6.3%)	0	0	0	0	
> 28 days of 2nd dose	17 (54.8%)	10 (62.5%)	5 (62.5%)	1 (100%)	2 (40%)	2 (66.7%)	
Duration from vaccine	56 (14–303)	50 (13–183)	22 (15–195)	85	31 (14–88)	60 (17–126)	** < 0.001**
Diagnostic CT^a^	5 (16.1%)	7 (43.8%)	6 (42.9%)	1 (100%)	1 (20%)	1 (33.3%)	0.15
Diagnostic swab	12 (38.7%)	4 (25%)	4 (28.6%)	0	2 (40%)	0	0.68
Diagnostic symptoms^b^	31 (100%)	16 (100%)	13 (92.9%)	1 (100%)	5 (100%)	3 (100%)	0.54
Fever	28 (90.3%)	16 (100%)	11 (78.6%)	1 (100%)	4 (80%)	3 (100%)	0.43
Headache	24 (77.4%)	13 (81.3%)	10 (71.4%)	1 (100%)	3 (60%)	2 (66.7%)	0.90
Myalgia	26 (83.9%)	16 (100%)	10 (71.4%)	1 (100%)	3 (60%)	3 (100%)	0.17
Ageusia	9 (29%)	5 (31.3%)	2 (14.3%)	0	2 (40%)	0	0.65
Anosmia	10 (32.3%)	4 (25%)	4 (28.6%)	0	2 (40%)	0	0.81
Cough	23 (74.2%)	13 (81.3%)	10 (71.4%)	0	4 (80%)	2 (66.7%)	0.61
Diarrhea	1 (3.2%)	3 (18.8%)	0	0	2 (40%)	1 (33.3%)	**0.04**
Rhinorrhea	9 (29%)	5 (31.3%)	4 (28.6%)	0	2 (40%)	1 (33.3%)	0.98
Sore throat	11 (35.5%)	7 (43.8%)	5 (35.7%)	1 (100%)	2 (40%)	2 (66.7%)	0.72
Dyspnea	11 (35.5%)	11 (68.8%)	8 (57.1%)	1 (100%)	1 (20%)	0	0.06
Care site treatment							
Home isolation	26 (83.9%)	9 (56.3%)	10 (71.4%)	0	3 (60%)	2 (66.7%)	
Oxygen therapy	0	1 (6.3%)	0	0	0	0	0.54
Hospital admission	2 (6.5%)	4 (25%)	3 (21.4%)	1 (100%)	1 (20%)	1 (33.3%)	
ICU admission	3 (9.7%)	2 (12.5%)	1 (7.1%)	0	1 (20%)	0	

Only significant values should be in boldface (*P* value was considered significant if < 0.05)

^a^CORAD>3

^b^symptoms suggestive of COVID-19 infection

Based on the current study, the predictors for post-vaccine COVID-19 infection included prior infection with odd’s ratio (OR) was 3.54, type of vaccine (AstraZeneca) with OR was 2.13, and partially vaccinated subjects with OR was 2.56 (Table 8).

**Table 8:** Regression analysis for prediction of post-vaccine COVID-19 infection (*N* = 70) among all vaccinated participants (*N* = 369)

	Odd’s ratio	95% CI	*P* value
Age	1.02	0.99–1.05	0.06
Marital state (married)	0.93	0.41–2.09	0.07
Occupation (employee)	3.14	1.62–6.08	0.06
Hypertension	3.84	0.98–15.03	0.66
Diabetes mellitus	1.45	0.26–7.92	0.99
Cardiac diseases	0.98	0.34–2.09	0.34
Prior infection	**3.54**	**1.89**–**6.67**	**0.01**
Type of vaccine (AstraZeneca)	**2.13**	**1.14**–**3.99**	**0.01**
Partially vaccinated	**2.56**	**1.16**–**5.62**	** < 0.001**

Only significant values should be in boldface (*P* value was considered significant if < 0.05). *COVID-19* coronavirus disease-19, *CI* confidence interval

## Discussion

In the current study of prevalence and characteristics of infection breakthrough after COVID-19 vaccination, we declare that 18.97% (70 patients) of the 369 subjects enrolled in the study had post-vaccination and infection breakthrough. Students were the most risk group representing 18.6% of the study subjects. Hypertension, diabetes, and cardiac diseases were the most comorbidities associated with a prevalence of 15.7%, 8.6%, and 4.3% respectively. Prior to COVID-19, confirmed infection was present in 42.9% of the study group. Two peaks of infection breakthrough were observed; after the second dose of vaccination by 28 days representing 52.7% of cases and between 14 and 28 days of the first dose of vaccination representing 27.1% of cases with overall median days from vaccination dose 41 days and range (14–303) days. We observed that infection breakthrough usually does not need hospital admission in only 19 patients. Seven of them needed intensive care unit admission. AstraZeneca vaccine type was the most common vaccine type associated with the occurrence of infection breakthrough which occurred in 44.3% of subjects.

Although up-to-date studies prove the efficacy and safety of COVID-19, vaccine infection breakthrough is not uncommon [8].

The possible etiology of infection breakthrough after COVID-19 vaccination is immunosuppression due to comorbidity or regular use of immune suppressant drugs such as steroids or after organ transplantation. It is acquiring infection a few days before vaccination or before well-established immunity or insufficient immune response and production of antibodies against the SARS-COV-2 virus [9]. Mutation, variants of concern (VOC), and vaccine escape are among other etiological causes of the infection breakthrough after COVID-19 vaccination [10].

The exact duration of immunity following COVID-19 vaccination is still being assessed. Some of COVID-19 infections could be due to waning immunity and antibody titers [11].

Improper practice of vaccination and storage is a concern in the etiology of infection breakthroughs reported in some studies [12].

High viral load and pandemic fatigue are among the factors that can influence and participate in infection breakthrough. Genetic factors are another possibility for infection breakthrough incidence after COVID-19 vaccination. Studies reported that the Black and Ethnic Minority (BAME) population is affected disproportionately by the SARS-COV-2 virus [12].

In agreement with our results, Baltas and his colleagues in the study of infection breakthrough after COVID-19 infection reported 116 of 119 cases developed COVID-19 post-first vaccination dose (median, 14 days) [13].

In accordance with our results in the study of infection breakthrough after COVID-19 vaccination in healthcare workers, Krishna B and her colleague reported an increased incidence of diabetic patients and patients with hypertension in 4.5% and 6.1% of all cases [14].

In disagreement with other results, Hacisuleyman E and his colleagues in a cohort study to assess the incidence of infection breakthrough after COVID-19 vaccination, only two women of his 417-study group developed infection breakthrough. We relate the resulting discrepancy to different population groups and short follow-up periods in his study [15].

As the results of our study, a study by Sharma and her colleagues where 11,197 infection breakthrough cases were followed up only 2080 cases needed hospitalization and were of mild to moderate severity [16].

In agreement with our results, Kustin and her colleagues enrolled 149 infection breakthrough patients after the vaccine’s first dose and 247 patients where infection occurred after the second dose and reported that 46 of the patients were breakthroughs occurred after the second dose after more than 20 days and 133 patient out of 247 were infection occurred after the first dose was within 7–14 days of vaccination [17].

In concomitant with our results, Shamier and his colleagues enrolled 161 breakthrough infection health co-workers after fully vaccinating 136 of them. They were symptomatic but none of them needed hospitalization. But the AstraZeneca vaccine contrary to our results was the least to cause infection breakthrough among all other vaccine types. We contribute this discrepancy to the limited number of health coworkers who received the AstraZeneca vaccine (932 subjects out of 22,169 health coworkers) [18].

In the context of our results, there is a study by Bergwerk and his colleagues which has similar results. Among 1497 healthcare workers with two vaccine dose 39 breakthrough infections were documented. Most breakthrough cases were mild or asymptomatic although 19% had persistent symptoms (> 6 weeks) [19].

In agreement with our results, Brunvoll and his colleagues enrolled 360 infections, breakthrough patients, after being fully vaccinated and reported that there was female predominance (73.1 of the cases), and the most reported symptoms were dyspnea and fatigue in 11.1% and 30.6% of patients respectively in the follow up of the patients. Four cases required hospitalization [20].

## Conclusion

Post-vaccination infection and breakthrough after COVID-19 vaccination is not uncommon. Most cases are mild and do not require hospitalization. All types of vaccines tested in the current study offer adequate immunity and guard against severe COVID-19 infection. We encourage the current global policy of full vaccination.

## Data Availability

The datasets analyzed during the current study are available upon request.

## Abbreviations

COVID-19: Corona virus disease 2019
FDA: Food and Drug Administration
HRCT: High-resolution computed tomography
MSCT: Multi-slice computed tomography
PCR: Polymerase chain reaction
SARS-CoV-2: Severe acute respiratory syndrome coronavirus-2
